# Position Fingerprint-Based Beam Selection in Millimeter Wave Heterogeneous Networks

**DOI:** 10.3390/s17092009

**Published:** 2017-09-01

**Authors:** Zufan Zhang, Yanbo Chen

**Affiliations:** School of Communication and Information Engineering, Chongqing University of Posts and Telecommunications, Chongqing 400065, China; zhangzf@cqupt.edu.cn

**Keywords:** millimeter wave, multi-cell, heterogeneous networks, position fingerprint matching, beam selection

## Abstract

The traditional beam selection algorithms determine the optimal beam direction by feeding back the perfect channel state information (CSI) in a millimeter wave (mmWave) massive Multiple-Input Multiple-Output (MIMO) system. Popular beam selection algorithms mostly focus on the methods of feedback and exhaustive search. In order to reduce the extra computational complexity coming from the redundant feedback and exhaustive search, a position fingerprint (PFP)-based mmWave multi-cell beam selection scheme is proposed in this paper. In the proposed scheme, the best beam identity (ID) and the strongest interference beam IDs from adjacent cells of each fingerprint spot are stored in a fingerprint database (FPDB), then the optimal beam and the strongest interference beams can be determined by matching the current PFP of the user equipment (UE) with the PFP in the FPDB instead of exhaustive search, and the orthogonal codes are also allocated to the optimal beam and the strongest interference beams. Simulation results show that the proposed PFP-based beam selection scheme can reduce the computational complexity and inter-cell interference and produce less feedback, and the system sum-rate for the mmWave heterogeneous networks is also improved.

## 1. Introduction

The rapid development of mobile services and the fast growth in the ownership of intelligent terminals demand an exponential growth in the wireless data rate. Compared with the fourth generation mobile communication (4G) system, the next generation mobile communication (5G) system is required to provide at least a 1000 times growth in capacity [1]. To achieve this goal, a large number of under-utilized millimeter wave (mmWave) bands (i.e., 30–300 GHz) have received much attention and mmWave wireless communication is considered to be a significant technique for 5G [2,3,4]. The shorter wavelength of the mmWave signal allows a base station (BS) to deploy tens or even hundreds of antennas in a relatively compact space, which easily caters for the massive Multiple-Input Multiple-Output (MIMO). Furthermore, massive MIMO can effectively increase the data rate as well as link reliability [5], and improve the energy efficiency and the spectral efficiency by three orders and 1–2 orders of magnitude respectively [6]. The combination of mmWave and massive MIMO can effectively overcome their respective shortcomings while their benefits can be sufficiently exploited. The latest research results have fully demonstrated the potential of mmWave massive MIMO for 5G wireless system [3,4].

Considering serious propagation and penetration losses that mmWave suffers, directional beamforming (BF) is considered to be a crucial technique to compensate for the high path loss in mmWave networks [7,8,9]. The BF technology can determine the best beam direction which is formed by multiple antenna elements for user equipments (UEs) to maximize the transmission rate and improve the energy efficiency. The traditional mmWave beam selection algorithms are all based on estimating the perfect channel state information (CSI), which require accurate channel estimation and CSI feedback to the BS [10]. However, the difficulty in capturing the real-time CSI for UEs in mmWave transmission systems greatly limits the traditional methods. In addition, the involved exhaustive beam search induces a large overhead and imposes a heavy computational burden on the system. Therefore, the new channel estimation algorithms for mmWave cellular systems have been developed in recent years. An adaptive compressed sensing (CS) based algorithm that efficiently estimates the parameters of mmWave channels was designed in [11]. By adopting a temporally correlated mmWave channel model, [12] presented two CS algorithms that exploit the temporal correlation to reduce the complexity of sparse channel estimation. In addition, the beam searching process can be accelerated with position or direction estimation. The authors of [13] presented an efficient method that capitalizes on the exchange of position information between the network nodes to design their BF and combining vectors. In [14], the concept of radar aided mmWave vehicular communication was introduced, and two protocols for beam searching in a vehicle-to-infrastructure (V2I) scenario were proposed. The simulation results confirmed that the main direction of arrival for the radar and the communication signals are similar, and the radar can be a useful source of side information that helps configure the mmWave V2I link.

In this paper, we propose a position fingerprint (PFP)-based mmWave multi-cell cooperation beam selection scheme, which needs less feedback and avoids high computational complexity caused by an exhaustive search, meanwhile suppressing inter-cell interference in mmWave heterogeneous networks. In the proposed scheme, the fingerprint database (FPDB) contains each fingerprint point’s PFP, the corresponding best beam ID and the strongest interference beam IDs from adjacent cells. The optimal beam and the strongest interference beams are determined through matching the current PFP of the UE with the PFP in the FPDB, and the orthogonal codes are allocated to the optimal beam and the strongest interference beams to reduce the inter-cell interference. This scheme can not only simplify the process of beam selection, but can also avoid the process of CSI estimation and largely reduce the information feedback. In addition, it can effectively suppress the interference among beams caused by adjacent cells. Simulation results show that the proposed scheme can achieve the near-optimal sum-rate performance and effectively avoid the beam interference caused by adjacent cells in cellular mmWave heterogeneous networks with densely deployed small cells.

The rest of this paper is organized as follows. In Section 2, we present an overview of the related work. Section 3 provides the proposed system model. The proposed PFP-based mmWave multi-cell beam selection scheme is presented in Section 4 and its performance is analyzed in Section 5. Section 6 concludes this paper.

## 2. Related Work

For the mmWave massive MIMO transmission, the communication industry has proposed a variety of BF and beam selection schemes, which mainly focused on the design of an mmWave beam codebook, beam selection algorithm and inter-beam interference suppression. These schemes are designed to effectively improve BF gain and reduce the link complexity, the number of radio frequency (RF) chains and the feedback. Medium Access Control (MAC) based BF protocols for mmWave transmission contain a beam codebook, the iterative search and the multiple sector ID capture (MIDC) [15,16], which involves exhaustive search and leads to high computational complexity. Random BF with partial CSI feedback is proposed to reduce the feedback of UE [17,18], which yields reasonable performance when the UE number of a cell reaches a certain threshold value. A new mmWave transmission scheme combines beamspace MIMO and beam selection [19,20], which makes full use of the sparsity of the mmWave beamspace channel matrix and then approaches the ideal system performance while reducing the hardware complexity and suppressing the inter-UE interference. The authors of [21] presented an enhanced spatial division multiple access (SDMA) scheme, in which the BS selects several beams that capture the channel’s main lobe, to approximate the original high-dimensional channel, thus to reduce the feedback. This scheme overcomes the difficulty in the downlink CSI acquisition for an mmWave massive MIMO system, but still needs to consider the computational complexity and the feedback design.

Existing beam selection algorithms are mainly based on the magnitude maximization selection (MM-S) [19]. However, it may result in the waste of some RF chains, and suboptimal performance in received signal-to-interference-plus-noise ratio (SINR) and capacity. For this reason, [18,20] proposed maximizing the SINR selection (MS-S) and capacity-maximization selection (MC-S). In the MS-S, UE scheduling and beam selection are performed by feeding back each UE’s maximum SINR to the BS, then the BS selects one beam with the maximum SINR for each UE. The MC-S algorithm is similar to the MS-S, whose main objective is the identification of a subset of beams with minimum capacity loss from the whole system. The MC-S can be performed with two different approaches: the decremental and incremental algorithm. Although the MM-S has a relatively low computational complexity, its performance is susceptible to the actual multi-path environments; MS-S and MC-S have relatively high computational complexity whereas their performances are closer to ideal performance in applications. Therefore, how to balance the performance and the computational complexity is a primary concern of the beam selection algorithm.

At present, the beam selection algorithms center on a single cell. Due to the high computational complexity caused by an exhaustive search, it is difficult to extend the existing beam selection algorithms to multi-cell. The traditional multi-cell coordinated BF technology is mostly concentrated on a simple model, i.e., three cells at most. In [22], a distributed iterative BF scheme was proposed, which exploits the network’s duality principle to jointly calculate the transmit-receive BF weights such that the desired receiving power of each UE is maximized while minimizing the accompanying interference. The current multi-cell coordinated BF algorithms are usually quite complex and impractical to apply to heterogeneous networks with densely deployed small cells. To the best of the authors’ knowledge, the beam selection is rarely studied in cellular heterogeneous networks with densely deployed mmWave small cells. This paper concentrates on the multi-cell cooperation beam selection scheme in cellular mmWave heterogeneous networks.

## 3. System Model

### 3.1. Millimeter Wave Heterogeneous Networks Model

**Figure 1:**
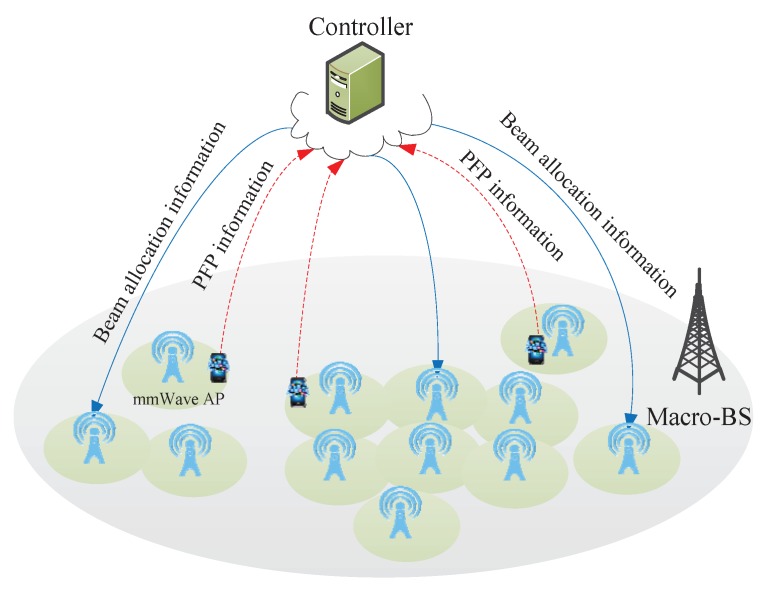
Millimeter wave heterogeneous networks.

The deployment of a large number of different types of cells, such as macro-, micro-, pico-, and femtocells, i.e., heterogeneous networks, is a key technique to achieve network densification and enhance networks’ system capacity, coverage performance and energy efficiency. In this paper, we introduce a mmWave heterogeneous network with densely deployed mmWave access points (APs) in the coverage of some macrocells, where the macrocells operate at microwave frequency band and the mmWave APs operate at mmWave frequency band which are deployed for traffic offloading [23]. We assume the UEs use dual band (mmWave/microwave frequency) for communication, which is a typical assumption for future 5G networks. The Time of Arrival (TOA) of the downlink macrocell special reference signal (M-CRS) is served as the PFP to indicate the current position of the UE. In Figure 1, mmWave APs and UEs are connected to a local controller that can be implemented as an AP controller for the mmWave APs.

### 3.2. Millimeter Wave Channel model

We consider a multi-cell Multiple-Input Single-Output (MISO) downlink system, where each mmWave AP employs *M* antennas and multi-RF chains to simultaneously serve *K* single-antenna UEs [19,20]. The received signal of the *k*th UE in the *i*th small cell is given by

(1) $$y_{i,k}=h_{i,i,k}^{H}w_{i}s_{i}+\sum_{j\neq i}^{N}h_{j,i,k}^{H}w_{j}s_{j}+n_{i,k},\;\forall i,k$$

where, *N* is the number of mmWave APs, $h_{j,i,k}\in C^{M\times 1}$ denotes the the mmWave sparse channel vector from the AP in the *j*th small cell to the *k*th UE in the *i*th small cell, $w_{i}\in C^{M\times 1}$ denotes the downlink BF vector used by the AP in the *i*th small cell, $s_{i}\in C$ denotes the information symbol intended for all *K* UEs in the *i*th small cell with $E\{|s_{i}{|}^{2}\}=1$, and $n_{i,k}$ is a circularly symmetric complex additive white Gaussian noise (AWGN) with mean zero and variance $\delta_{i,k}^{2}$. The receiving SINR of the *k*th UE in the *i*th small cell can be given by

(2) $${SINR}_{i,k}=\frac{|h_{i,i,k}^{H}w_{i}{|}^{2}}{\sum_{j\neq i}^{N}{\left|h_{j,i,k}^{H}w_{j}\right|}^{2}+\delta_{i,k}^{2}},\;\forall i,k$$

The system sum-rate maximization problem under per-AP power constraints can be formulated as follows:

(3) $$R=\underset{\left\{w_{i}\right\},\left\{\gamma_{i}\right\}}{max}\sum_{i=1}^{N}\alpha_{i}\log_{2}\left(1+\gamma_{i}\right)$$

(4) $$\begin{array}{cc} s.t. & {\left\|w_{i}\right\|}_{2}^{2}\leq P_{i},\forall i\\ & SINR_{i,k}\geq \gamma_{i},\forall i,k \end{array}$$

where, $\alpha_{i}$ is the weight and $P_{i}$ is the AP transmit power budget in the *i*th small cell.

**Figure 2:**
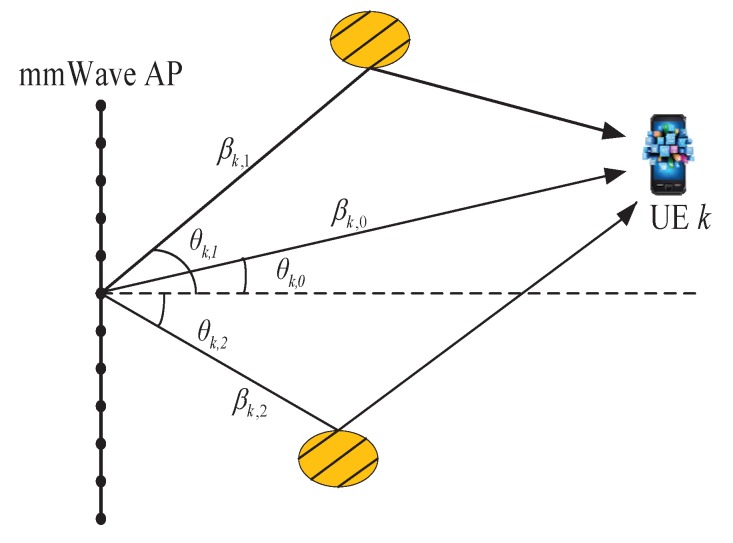
The Saleh-Valenzuela channel model (L=2).

Since the mmWave signal suffers from high path loss and limited scattering, spatial multiplexing is limited in the mmWave channel, and the channel becomes spatially sparse [17]. We consider a widely used Saleh-Valenzuela channel model to describe the mmWave channel [19,20]. For simplicity, we adopt a single-cell as an example to analyze a multi-UE MISO downlink, in which AP employs the typical uniform liner array (ULA) with *M* antennas and serves *K* single-antenna UEs. The channel vector $h_{k}$ of the *k*th UE is given by

(5) $$h_{k}=h_{k}^{\left(\mathrm{LOS}\right)}+h_{k}^{\left(\mathrm{MP}\right)}=\beta_{k,0}a\left(\theta_{k,0}\right)+\sum_{i=1}^{L}\beta_{k,i}a\left(\theta_{k,i}\right)$$

where, $h_{k}^{\left(\mathrm{LOS}\right)}$ is the line-of-sight (LOS) component of the *k*th UE with $\beta_{k,0}$ presenting the path gain, $h_{k}^{\left(\mathrm{MP}\right)}$ is the non-line-of-sight (NLOS) component of the *k*th UE and *L* is the total number of NLOS components, θ is the normalized angle of departure (AoD) of a path included in the channel vector $h_{k}$. $a\left(\theta \right)$ is the ULA steering vector given by

(6) $$a\left(\theta \right)=\frac{1}{\sqrt{M}}{\left[1,e^{-j\pi \theta },\cdots ,e^{-j(M-1)\pi \theta }\right]}^{T}$$

where, the normalized AoD θ∈[−1,1] is related to the physical AoD ϕ∈[−π/2,π/2] as

(7) $$\theta =\frac{2dsin(\phi )}{\lambda }$$

where, *d* is the distance between two adjacent antenna elements, λ denotes the carrier wavelength, and d/λ=0.5. Since we consider the MISO case and AoD matters, assuming that there exists a scatter or reflector at each AoD involved in the NLOS component given by Equation (5) to generate a transmission path from the mmWave AP to UE *k* at that AoD, the channel model is shown in Figure 2.

A key challenge in implementing an mmWave massive MIMO is that the conventional digital BF requires a dedicated RF chain for each antenna, resulting in expensive hardware costs and power losses. The digital-analog hybrid BF technique is an alternative method to overcome both high computational complexity of large array system and high hardware costs [24]. Yet the concept of beamspace MIMO has been recently proposed in pioneering work. By employing the discrete lens array (DLA) with negligible performance loss [25], a beamspace MIMO can transform the conventional spatial channel to the beamspace channel to capture the channel sparsity at mmWave frequencies. Therefore, the employed DLA is an M×M spatial discrete fourier transform matrix U, which contains the array steering vectors of *M* orthogonal directions covering the entire space as

(8) $$U={\left[a\left({\bar{\theta }}_{1}\right),a\left({\bar{\theta }}_{2}\right),\cdots ,a\left({\bar{\theta }}_{M}\right)\right]}^{H}$$

where, ${\bar{\theta }}_{m}=\frac{1}{M}\left(m-\frac{M+1}{2}\right),m=1,2,\cdots M$ are the predefined spatial directions. Then, the beamspace channel model can be represented by

(9) $$\bar{H}=\left[{\bar{h}}_{1},{\bar{h}}_{2},\cdots ,{\bar{h}}_{K}\right]=UH=\left[Uh_{1},Uh_{2},\cdots ,Uh_{K}\right]$$

where, ${\bar{h}}_{k}$ is the beamspace channel of the *k*th user. In Equation (9), the *M* rows (elements) of $\bar{H}\left({\bar{h}}_{k}\right)$ correspond to *M* orthogonal beams whose spatial directions are ${\bar{\theta }}_{1},{\bar{\theta }}_{2},\cdots ,{\bar{\theta }}_{M}$, respectively. Due to the number of dominant scatters being quite limited in the mmWave propagation environment [19], the beamspace channel enjoys a sparse structure. According to the sparse beamspace channel, we can select a small number of appropriate beams to reduce the dimension of a massive MIMO system without obvious performance loss, which is exactly the beam selection problem that needs to be solved.

## 4. PFP-Based mmWave Multi-Cell Beam Selection Scheme

We propose a PFP-based mmWave multi-cell cooperation beam selection scheme in mmWave heterogeneous networks with densely deployed small cells, which is based on the PFP of the UE. The wide coverage fingerprints are used to lock the optimal beam IDs and the corresponding strongest interference beam IDs for the current positions of the UEs. Thus, the optimal beam and the strongest interference beam can be determined through matching the current PFP of the UE with the PFP in the FPDB. Furthermore, if the strongest interference beams are simultaneously used by the adjacent cells to transmit data, the orthogonal codes are allocated to the optimal beam and the strongest interference beams to reduce the inter-cell interference. The FPDB is assumed to be formed and stored in the controller in the offline phase.

Due to the mmWave suffering from significant propagation loss, it is reasonable to consider only the UEs in the edge area of the cell suffering from the adjacent cell interference. Therefore, the UEs served by an mmWave AP can be divided into two categories: inner UEs and edge UEs, which are located in the inner area and edge area of a small cell, respectively. The fingerprint information of a small cell’s inner area only contains the service AP ID and the corresponding best beam ID, while the fingerprint information of a small cell’s edge area not only contains the service AP ID and the corresponding best beam ID, but also includes the adjacent mmWave AP IDs and the corresponding strongest interference beam IDs. In the proposed scheme, we use the downlink M-CRS’s TOA as a simple PFP for the UE’s position, and the number of macrocells is no less than three [26]. The reason for selecting the cell special reference signal (CRS) as the TOA measurement signal is that UE periodically performs synchronous tracking and Reference Signal Receiving Power (RSRP) measurement according to the downlink CRS in the LTE system. Then the TOA can be acquired via the process of UE’s synchronous tracking. Using CRS as the measurement signal will not increase the system overhead and the forward compatibility is guaranteed.

**Figure 3:**
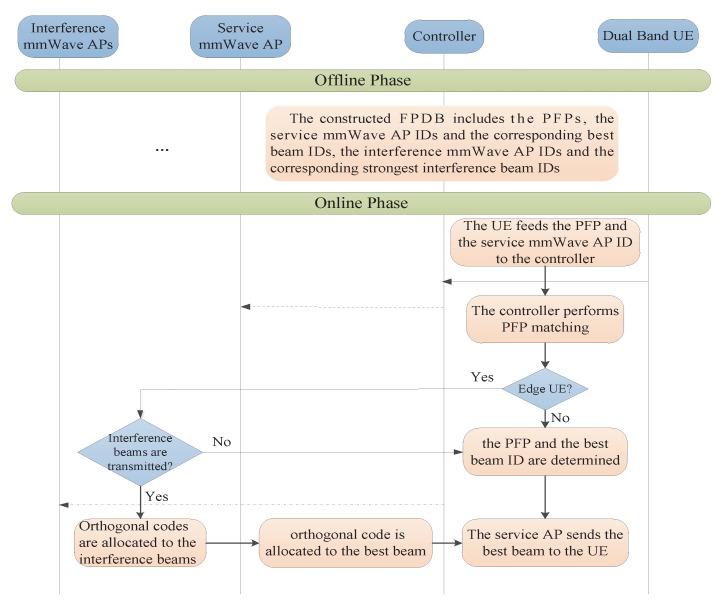
The proposed position fingerprint (PFP)-based multi-cell beam selection scheme.

The UE in networks periodically detects the TOAs of the downlink M-CRS transmitted by different macrocells and reports the test results to the local controller. Afterwards, the local controller finds the matched PFP in the PFDB and determines the optimal beam and the strongest interference beams for the UE according to the matched PFP. Other fingerprint techniques such as Time Difference of Arrival (TDOA), Receive Signal Strength (RSS) and Arrival of Angle (AOA) can be easily used by extending the proposed PFP-based scheme [27]. Figure 3 shows the general framework of the PFP-based beam selection for one mmWave AP-UE link, which will be explained in detail in the subsequent sub-sections.

### 4.1. The Offline FPDB Construction

All the mmWave AP coverage areas are densely divided into multi-mesh area and a fingerprint point is selected for each mesh area to represent all the UEs whose physical positions are in that area. There are *W* fingerprint spots in the inner area and *Q* fingerprint spots in the edge area of one small cell. Due to the coverage area of the mmWave AP being small, the number of the fingerprint points is not very large for each small cell, and the cost of establishing the FPDB is tolerable. Since the PFDB is set up in offline phase, the corresponding fare is not discussed in this paper.

**Figure 4:**
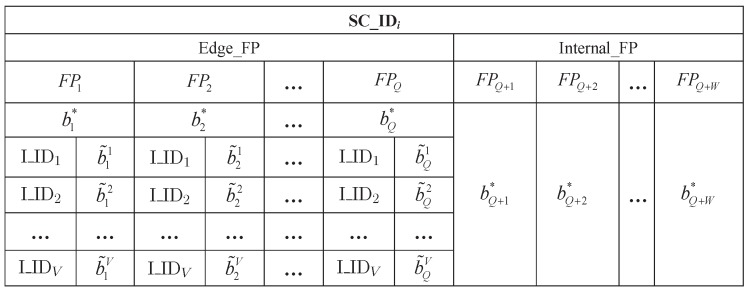
The fingerprint data block of small cell *i*.

The measurement is performed in each small cell by assigning the UE to each fingerprint spot and the measurement content includes the fingerprint spot’s PFP, the service mmWave AP ID and the corresponding optimal beam ID. If the fingerprint spot is in edge area of small cell, the adjacent mmWave AP IDs and the corresponding strongest interference beam IDs also need to be measured. Then, the measurement result is saved in the corresponding fingerprint data block, i.e., a set of all fingerprint information within an mmWave AP coverage area. Each fingerprint data block is divided into two parts, namely, the internal fingerprint data set referring to Internal_FP and the edge fingerprint data set referring to Edge_FP. Figure 4 shows the fingerprint data block of the small cell *i*, in which $\mathrm{SC}\_{\mathrm{ID}}_{i}$ is the ID of the small cell *i*, $FP_{q}\left(q=1,2,\cdots ,Q\right)$ and $FP_{w}\left(w=Q+1,Q+2,\cdots ,Q+W\right)$ are the edge PFP for fingerprint spot *q* and the inner PFP for fingerprint spot *w*, respectively, ${I\_\mathrm{ID}}_{v}\left(v=1,2,\cdots ,V\right)$ is an adjacent interference mmWave AP ID for a fingerprint spot, $b_{q}^{*}$ and ${\tilde{b}}_{q}^{v}$ are the optimal beam ID and the strongest interference beam ID corresponding to the adjacent interference mmWave AP *v* for $FP_{q}$ respectively, and $b_{w}^{*}$ is the optimal beam ID for $FP_{w}$.

During the offline FPDB construction phase, the UE of each fingerprint spot performs the widely used beam selection algorithm, i.e., MM-S to determine the optimal beam transmitted by the service mmWave AP and the strongest interference beams caused by the adjacent cells. Here we assume all the beams generated by the same mmWave small cell are orthogonal. Furthermore, the same beam serves multiple UEs through a time division multiplexing mechanism to avoid intra-cell interference.

The channel matrix of the fingerprint spot in a small cell can be obtained by the UE assigned to the fingerprint spot. Then, the conventional spatial channel is transformed into a beamspace channel by employing the DLA, as shown in Equations (8) and (9). Finally, the optimal beam is obtained through the MM-S. Furthermore, the strongest interference beams of the fingerprint spot are also determined by this method. In other words, both the optimal beam transmitted by the service mmWave AP and the strongest interference beams from the adjacent mmWave APs of each fingerprint spot are obtained by the MM-S. Note that only the edge fingerprint points need to determine the strongest interference beams.

The beam selection process is illustrated by taking edge PFP $FP_{q}$ as an example with the assumption that the fingerprint point *q* is in the edge area of the small cell *i*, the DLA employed by the small cell *i* is $U_{i}$, the employed beam IDs in each beamspace of different small cell are the same and can be expressed as *b*, $b\in \left\{1,2,\cdots ,M\right\}$. The beamspace channel matrix of the fingerprint spot *q* can be obtained by Equation (10). Then, the optimal beam is obtained by the MM-S, as shown by Equation (11):

(10) $${\bar{h}}_{i}\left(b\right)=U_{i}\left(b,:\right)h_{i,i,q},b\in \left\{1,2,\ldots ,M\right\}$$

(11) $$b_{q}^{*}=\underset{b\in \{1,2,\ldots ,M\}}{argmax}\;\left\{{\bar{h}}_{i}\left(b\right)\right\}$$

(12) $${\bar{h}}_{j}\left(b\right)=U_{j}\left(b,:\right)h_{j,i,q},b\in \left\{1,2,\ldots ,M\right\}$$

(13) $${\tilde{b}}_{q}^{j}=\underset{b\in \{1,2,\ldots ,M\}}{argmax}\;\left\{{\bar{h}}_{j}\left(b\right)\right\}$$

where, ${\bar{h}}_{i}$ is beamspace channel between the fingerprint spot *q* and the service mmWave AP in the small cell *i* , $b_{q}^{*}$ is the optimal beam ID for the fingerprint spot *q*. Similarly, the beamspace channel matrix between the fingerprint spot *q* and the interference mmWave AP in the small cell *j* can be obtained by Equation (12). Then, the strongest interference beam caused by the mmWave small cell *j* is also obtained by the MM-S. The formula is given by Equation (13).

### 4.2. The Online PFP Matching

The UE will perform low frequency band detection periodically in mmWave heterogeneous networks with densely deployed small cells. The detection content includes the macrocell IDs and the corresponding downlink M-CRS’s TOAs, i.e, the UE’s current PFP, and the detection result is fed back to the controller as well. Then, the controller matches the PFP fed back by the UE with that of the FPDB. During the matching process, the controller first determines the corresponding fingerprint data block according to the service mmWave AP ID involved in the feedback information. Afterwards, the PFPs in the fingerprint data block are matched one by one. Matching refers to identifying the best matched PFP in the fingerprint data block which has the smallest error with the UE’s current PFP, as shown by Equations (14)–(16):

(14) $$B_{\mathrm{FP}}=B_{\mathrm{Edge}\_\mathrm{FP}}\cup B_{\mathrm{Internal}\_\mathrm{FP}}$$

(15) $$FP_{i}=\left({\mathrm{TOA}}_{1},{\mathrm{TOA}}_{2},\cdots ,{\mathrm{TOA}}_{N}\right)\in B_{\mathrm{FP}},i\in \left\{1,2,\cdots ,W+Q\right\}$$

(16) $$FP_{i}^{*}=\underset{i\in \{1,2,\cdots ,W+Q\}}{\arg\min}\;\left(\left|f_{user}-FP_{i}\right|\right)$$

where, $B_{\mathrm{FP}}$ is the fingerprint data block of the UE’s service mmWave small cell, $B_{\mathrm{Edge}\_\mathrm{FP}}$ and $B_{\mathrm{Internal}\_\mathrm{FP}}$ are the data set of Edge_FP and Internal_FP respectively. $f_{user}$ is the UE’s current PFP, $FP_{i}$ is the PFP for fingerprint point *i*, ${\mathrm{TOA}}_{n}\left(n=1,2,\cdots ,N\right)$ correspond to the TOA of the *n*th M-CRS. $FP_{i}^{*}$ is the best-matched PFP. As a result, a matched PFP that is closest to the UE’s current PFP can be obtained.

After the matching process, the controller can further determine which fingerprint data set of the UE’s service small cell the matched PFP belongs to. If the matched PFP is in the Edge_FP, the UE can be judged as an edge UE, otherwise, it is an inner UE. When a UE is an inner UE, the controller only needs to notify the corresponding service mmWave AP to transmit the best beam to the UE according to the beam ID corresponding the matched PFP. If the UE is an edge UE, we need to judge whether the strongest interference beams are selected by the adjacent cells as the transmission beams before informing the service mmWave AP to transmit the best beam. When the interference beams are transmitted, it is necessary to allocate orthogonal codes to the optimal beam and the strongest interference beams. Therefore, the UE can distinguish the best target beam from the service mmWave AP and the strongest interference beams transmitted by the adjacent mmWave APs to achieve the purpose of interference cancellation.

Considering the characteristic of mmWave transmission, we design a method to solve the inter-cell beam interference problem in heterogeneous networks with densely deployed mmWave small cells. Due to the serious loss of the mmWave transmission, the inter-cell interference mainly comes from the adjacent small cells, and only the edge UEs suffer from the serious inter-cell interference. For this reason, the conception of taking advantage of the orthogonality of the PN codes in code division multiple access (CDMA) technology is adopted here to reduce interference. By adaptively assigning the PN codes to the interference beams and the optimal target beam, different beams are identified by different PN codes and the independent beams are formed in the spatial channel, which improves the interference suppression performance and the system performance.

**Figure 5:**
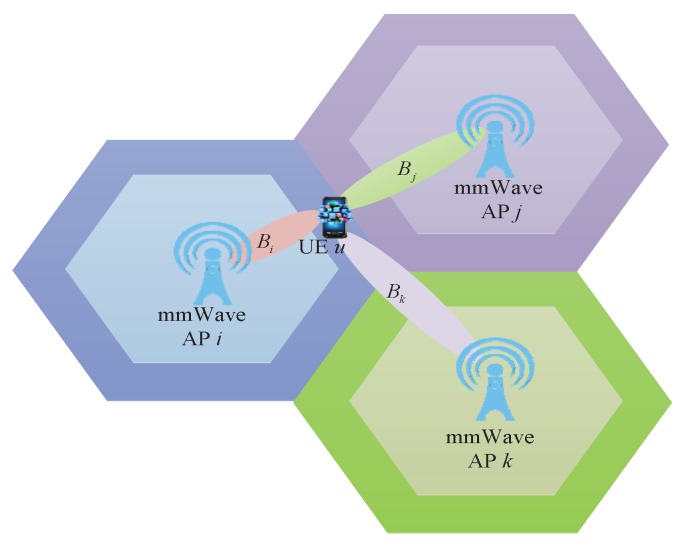
The best beam and the strongest interference beams of the edge user equipment (UE).

For the sake of simplicity, taking the edge UE *u* as an example, three small cells are used for beam interference coordination, as shown in Figure 5, $B_{i}$ is the optimal target beam, $B_{j}$ and $B_{k}$ are the strongest interference beams transmitted by the adjacent mmWave AP *j* and *k* respectively. If two or more beams belonging to $\left\{B_{i},B_{j},B_{k}\right\}$ are simultaneously selected by their respective small cells as the best transmission beams, the orthogonal codes need to be adaptively allocated to these beams before transmission so that the UE *u* can distinguish them. As previously described, the fingerprint information of Edge_FP includes not only the service mmWave AP ID and the corresponding best beam ID, but also the adjacent mmWave AP IDs and the corresponding strongest interference beam IDs. During the process of beam selection, the edge UE *u* first reports the current PFP and the service mmWave AP *i* to the controller, then the controller finds the matched PFP in the fingerprint data block of small cell *i* for UE *u*, so the optimal beam transmitted by the service mmWave AP *i* and the strongest interference beams caused by adjacent mmWave AP *j* and *k* can be determined.

## 5. Simulation Analysis

### 5.1. Contrastive Scheme Description

The following are scheme settings. Among them, Scheme 3 and Scheme 4 are the proposed PFP-based beam selection in this paper.

- Scheme 1: Fully digital BF system
  Each antenna corresponds to a RF chain, all beams are used to send data, and it does not involve the beam selection algorithm.
- Scheme 2: MM-S scheme
  Exploiting the sparsity of the mmWave channel matrix, the channel with the largest energy is selected, and an optimum transmission beam is determined for each UE.
- Scheme 3: TOA PFP-based noncooperation beam selection scheme
  The UE’s current TOA PFP is matched with the PFP in the FPDB to conduct the beam selection process and the FPDB includes only the UE’s PFP and the corresponding best beam ID. As a result, it is mainly applied to a single cell. When it is used in a multi-cell scenario, inter-cell beam interference is not taken into account.
- Scheme 4: TOA PFP-based multi-cell cooperation beam selection scheme
  UE’s current TOA PFP is matched with the PFP in the FPDB to conduct the beam selection process. In the matching process, the optimal beam and the strongest interference beams of the UE are obtained and the orthogonal codes are allocated to them before transmission, so as to eliminate the inter-cell beam interference.

### 5.2. Simulation Parameters

Simulation scenarios are in mmWave cellular heterogeneous networks with densely deployed small cells. The networks include 3 macrocells and 50 small cells that are evenly distributed. In addition, the macrocells and the small cells operate at microwave frequencies and mmWave frequencies respectively, and UEs’ positions are subject to Poisson distribution in the networks. We refer to the IEEE 802.11ad BF protocol and select the 60GHz as the carrier frequency of the mmWave AP. Specific parameters are summarized in Table 1 and Table 2. The path loss models are [28,29]:

(17) $$PL_{M}=22.5+39.38\log_{10}\left(d_{M}\right)\left(dB\right)$$

(18) $$PL_{S}=A+20\log_{10}\left(f_{c}\right)+10n\log_{10}\left(d_{S}\right)\left(dB\right)$$

where, $PL_{M}$ and $PL_{S}$ are the path losses of macrocell and small cell respectively, $f_{c}$ is the carrier frequency in GHz, $d_{M}$ and $d_{S}$ are the distance from UE to macrocell and small cell respectively, *A* is the attenuation value, and *n* is the path loss exponent.

For the spatial mmWave channel of UE *k* in a small cell, we have: one LOS component with L=2 NLOS components; $\beta_{k,0}∼\mathrm{CN}\left(0,1\right),\beta_{k,l}∼\mathrm{CN}\left(0,10^{-1}\right)$ for l=1,2; $\theta_{k,0}$, $\theta_{k,l}$ follow the i.i.d. uniform distribution within $\left[-\frac{1}{2},\frac{1}{2}\right]$ [19].

### 5.3. Simulation Results Analysis

**Figure 6:**
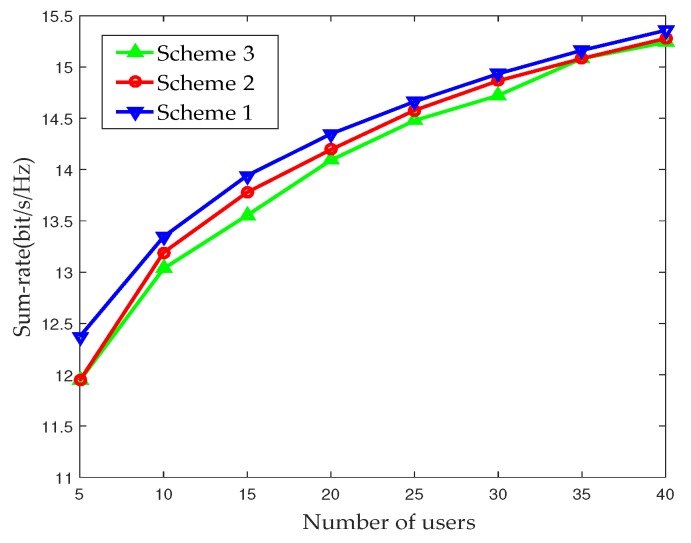
Sum-rate with respect to the number of UEs in single-cell.

Figure 6 shows the relationship between the sum-rate and the number of UEs in single-cell scenario. Compared with the fully digital BF and MM-S, the sum-rate of the proposed PFP-based scheme deteriorates slightly. For the proposed PFP-based scheme, the performance is greatly affected by the accuracy of the UE’s PFP measurement. When the error between the current PFP and the actual PFP is large, the matching result of the controller will be wrong, and the selected beam is not the best beam for the current UE. Although the PFP-based scheme has a slight performance decrease, it can avoid the computational complexity caused by the exhaustive search. In the proposed scheme, the UE only needs to feed back the simple PFP, and the controller matches the current PFP of the UE with that of the FPDB to obtain the optimal beam. The MM-S enjoys better performance but incurs prohibitively high computational complexity with $\left({}_{M}^{K}\right)$ searches. Furthermore, as the number of small cells increases, the computational complexity grows exponentially in mmWave heterogeneous networks with densely deployed small cells. In contrast, the proposed PFP-based scheme performs the beam selection by simply comparing the current PFP of the UE with the PFP in FPDB without an exhaustive search. Obviously, the beam selection process of the proposed PFP-based scheme can be greatly simplified.

**Figure 7:**
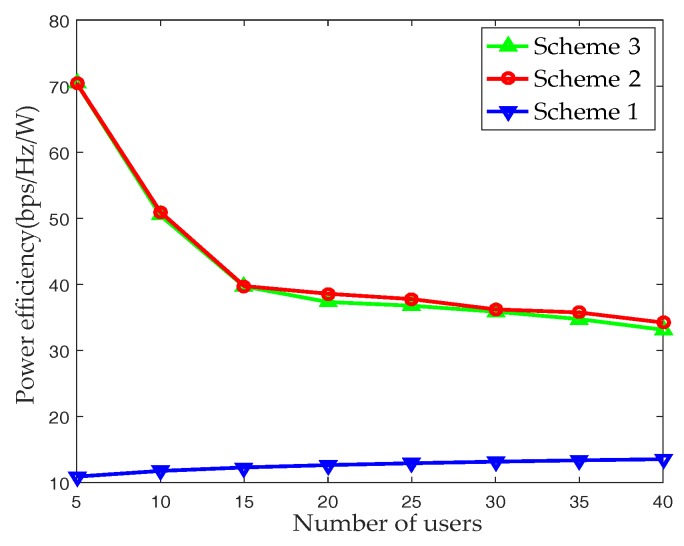
Power efficiency with respect to the number of UEs in single-cell.

Figure 7 shows the relationship between power efficiency and the number of UEs. The power efficiency ς is modeled as [20]:

(19) $$\varsigma =\frac{R}{\rho +N_{RF}P_{RF}}\left(bps/Hz/W\right)$$

where, ρ is the transmission power, $P_{RF}$ is the power consumed by RF chain. We adopt the practical values $P_{RF}=34.4$ mW and ρ=32 mW (15 dBm). It can be seen that the power efficiency of the fully digital BF scheme remains essentially constant and maintains a low level as the number of UEs increases. In Scheme 1, the number of transmitted beams is fixed and equals to the number of the antennas. For Scheme 2 and Scheme 3, the AP selects the appropriate beams for transmission according to their respective feedback information, and the number of transmission beams is not fixed and less than the number of antennas. Therefore, the power efficiency of fully digital BF is significantly lower than that of Scheme 2 and Scheme 3, while the power efficiency of Scheme 2 and Scheme 3 are basically the same.

**Figure 8:**
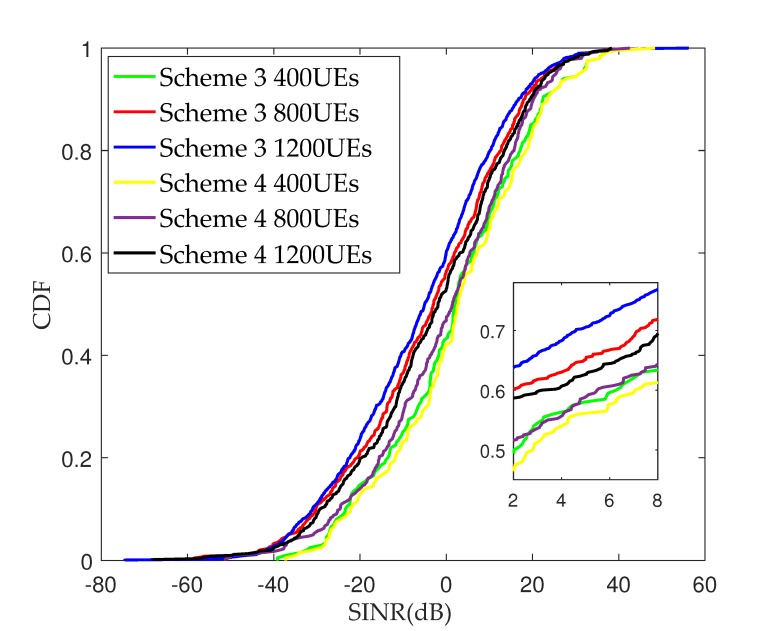
Signal-to-interference-plus-noise ratio (SINR) cumulative distribution functions (CDFs) with various numbers of UEs.

Figure 8 plots the SINR cumulative distribution function (CDF) for Scheme 3 and Scheme 4 in cellular mmWave heterogeneous networks with densely deployed small cells. It can be seen that, with the increasing in the number of UEs, the inter-cell beam interference becomes greater and the value of SINR in Scheme 4 is significantly improved compared to that in Scheme 3. The reason for this is that the orthogonal codes are used to distinguish the best beam and the strongest interference beams generated by the adjacent cells for the edge UE in Scheme 4, which is a benefit of interference cancellation. Due to the serious path loss of mmWave transmission, the interference beams received by UEs mainly come from the adjacent cells, and only the edge UEs suffer from the interference. When the number of UEs is 400, the performance of Scheme 3 and Scheme 4 tends to be consistent. Since a small number of UEs leads to a few transmission beams in each cell, and the possibility of inter-cell beam interference is tiny, the performance of the two schemes appears basically consistent. It can be concluded that the PFP-based multi-cell cooperation beam selection scheme can effectively avoid inter-cell interference in cellular heterogeneous networks with densely deployed mmWave small cells. With an increasing in the number of UEs, the advantage multi-cell cooperation is more obvious.

**Figure 9:**
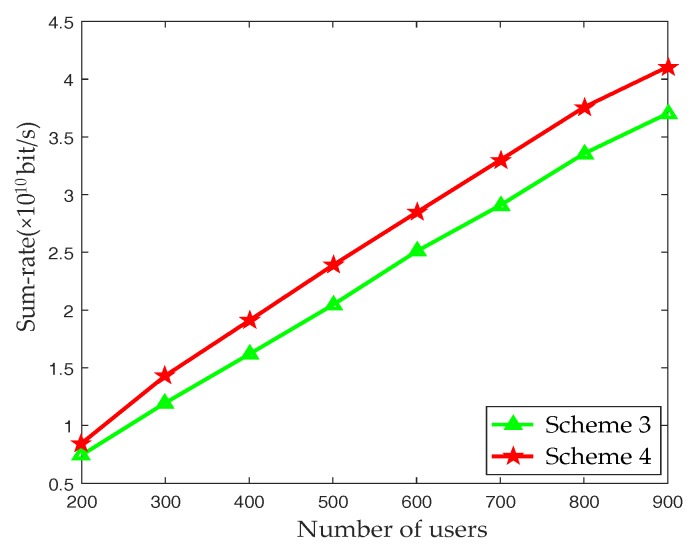
System sum-rate with respect to the number of UEs in multi-cell.

Figure 9 depicts the trend of the system sum-rate over the number of UEs of Scheme 3 and Scheme 4 in cellular mmWave heterogeneous networks with densely deployed small cells. It can be observed that the PFP-based multi-cell cooperation beam selection scheme can effectively improve the system sum-rate of the networks compared with the PFP-based noncooperation beam selection. The performance of Scheme 4 is much better than that of Scheme 3 with an increasing number of UEs. For Scheme 4, the optimal beam and the strongest interference beams of the edge UE are determined, and the orthogonal codes are allocated to the beams to reduce the inter-cell beam interference and improve the system sum-rate.

In summary, the PFP-based beam selection scheme can effectively select the appropriate beam for the UE and avoid the CSI feedback, while the computational complexity of the exhaustive search can be eliminated. In addition, especially in the mmWave heterogeneous networks with densely deployed small cells, the proposed scheme can effectively restrain the influence of beam interference caused by adjacent cells and improve the system performance.

## 6. Conclusions

In this paper, the beam selection scheme and the multi-cell cooperation BF scheme based on PFP are proposed in cellular mmWave heterogeneous networks with densely deployed small cells by taking advantage of the transmission characteristics of mmWave. Compared with the existing beam selection algorithms, the proposed PFP-based scheme can avoid the shortcomings of the computational complexity caused by exhaustive search algorithm while reducing the feedback. In particular, the proposed multi-cell cooperation scheme can effectively reduce the inter-cell beam interference in mmWave heterogeneous networks, and then improve the system sum-rate.

## Figures and Tables

**Table 1:** Simulation parameters No. 1.

Parameters	Macrocell	mmWave Small Cell
Coverage radius	1500 m	50 m
Transmit power	46 dBm	15 dBm
Antenna No.	-	64
RF chains	-	32
Attenuation value, *A*	-	$A_{\mathrm{LOS}}=32.5$, $A_{\mathrm{NLOS}}=45.5$
Path loss exponent, *n*	-	$n_{\mathrm{LOS}}=2.0$, $n_{\mathrm{NLOS}}=1.4$
Frequency band	2.5 GHz	60 GHz
bandwidth	20 MHz	1 GHz

**Table 2:** Simulation parameters No. 2.

Parameters	Value
Low frequency detection period	200 ms
PFP length	3
Fingerprint Matching Period	1 s
Noise density	−204 dBm/Hz
